# Symptomatic Supraventricular Tachycardia Resistant to Adenosine Therapy in a Patient with Chronic Theophylline Use

**DOI:** 10.1155/2021/2806193

**Published:** 2021-03-27

**Authors:** Seyedeh Maryam Hosseini, Muhammad Ajmal, Ranjith Shetty

**Affiliations:** ^1^University of Arizona College of Medicine, USA; ^2^Carondelet Medical Group, USA

## Abstract

Theophylline is a potent adenosine receptor antagonist with indirect adrenergic effects that can lead to arrhythmias and metabolic abnormalities such as hypokalemia. Therapeutic toxicity cases have declined over the years mainly due to decreased recommended therapeutic doses and overall decreased usage of this medication due to newer available COPD treatment options. We present a clinical case of symptomatic supraventricular tachycardia resistant to adenosine therapy in a patient with theophylline use. This case highlights the importance of comprehensive medication review in acute settings to aid in identifying the underlying etiologies and initiating prompt treatments. It also signifies the importance of reviewing chronic medications in each outpatient visits to ensure continued indication for their use and be able to change them to newer agents per guidelines whenever possible.

## 1. Introduction

Theophylline is well known for its bronchodilation effects. It is used for pulmonary obstructive disease, severe bradycardia, apnea, and asthma in certain premature and pediatric patients. It is also associated with adrenergic effects that can predispose patients to arrhythmias. We present a case of symptomatic supraventricular tachycardia unresponsive to adenosine.

## 2. Clinical Case Description

A 62-year-old male with a past medical history significant for hypertension, heart failure with persevered ejection fraction (HFpEF), alcohol abuse, chronic obstructive pulmonary disease (COPD) on chronic theophylline presented with epigastric abdominal pain, and vomiting after one day of heavy alcohol drinking. He denied hematemesis, melena, fall, and change in mental status. He was admitted with protocol in place for acute alcohol toxicity and alcohol withdrawal care. His initial presentation was significant for sinus tachycardia that resolved with fluid resuscitation. He had negative troponin values, positive elevated serum alcohol levels, metabolic acidosis, and normal hepatic function enzymes with no signs of acute infection or bleeding.

**Figure 1:**
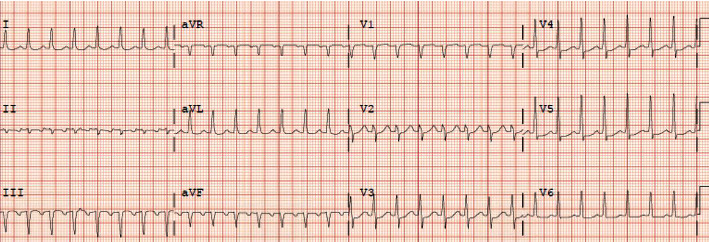
Initial EKG showing supraventricular tachycardia with heart rate of 181 beats per minute.

**Figure 2:**
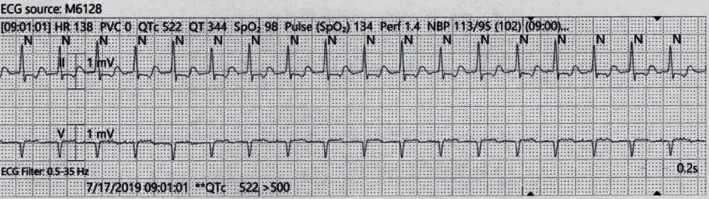
Patient's rhythm strip showing no improvement in response to adenosine.

**Figure 3:**
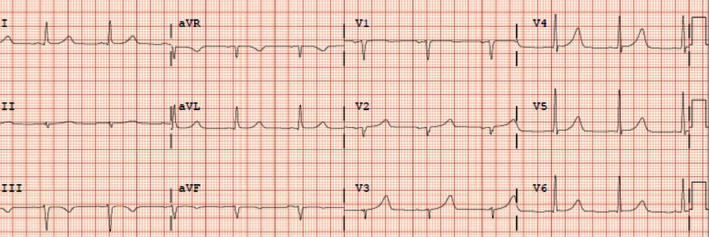
Patient's EKG after completion of amiodarone bolus showing resolution of supraventricular tachycardia.

On the second day of admission, the patient developed shortness of breath and palpitations. EKG showed supraventricular tachycardia, atrioventricular tachycardia nodal reentrant type, with a heart rate of 181 beats per minute (Figure 1). 6 milligram (mg) of adenosine was administered as rapid IV push but did not terminate the arrhythmia, and the next dose of 12 mg of adenosine rapid IV push was administered which was also unsuccessful (Figure 2). Because of the failure of adenosine to terminate arrhythmia and decreasing blood pressure measurements most likely secondary to ongoing SVT, cardioversion for unstable SVT was considered; however, patient regained hemodynamic stability quickly with administration of intravenous bolus of fluids as well as amiodarone 150 mg which was able to terminate the arrhythmia (Figure 3). His blood pressure improved from 85/60 mmHg to 122/74 mmHg after arrhythmia termination. Patient remained responsive, alert, and oriented and only complained of palpitations while having SVT. Serum theophylline level was found to be 4.67ug/mL (Table 1).

Patient's theophylline was stopped. He was started on tiotropium, an inhaled long-acting muscarinic antagonist as well as rescue inhaler albuterol, short acting beta agonist, for his COPD management. Patient was monitored in Coronary Care Unit for 24 hours and remained symptom free. He was transferred to medicine unit and discharged after two additional days of hospitalization. At discharge patient was recommended to take his new inhaler medications for COPD while stopping theophylline. Patient was discharged in stable condition with no further need or indication of amiodarone or any other antiarrhythmic drug.

## 3. Discussion

Theophylline metabolism and kinetics have a wide variation as a function of age. It is rapidly absorbed, metabolized by the liver, and excreted by the kidney. Toxic levels are seen mainly in serum concentrations of above 20 *μ*g/mL, but symptoms of toxicity can be seen at even lower end of therapeutic levels in some older patients and in those with chronic theophylline use such as in our case [1]. Theophylline effects are well explained by its mechanism of action. It is a potent adenosine receptor antagonist. In a case series study done in Massachusetts Children's Hospital, theophylline intoxication was shown to be associated with increased catecholamine serum levels in humans [2]. This was also shown on animal studies [3]. Theophylline is an antagonism of all adenosine receptor types, A1, A2A, A2B, and A3 which leads to increased norepinephrine release causing overall indirect adrenergic effects and thus metabolic abnormalities such as hypokalemia, hyperglycemia, and metabolic acidosis. It also predisposes to arrhythmias and can cause hypotension that is likely mediated by not only arrhythmias but also by beta adrenergic vasodilatory mechanism. Theophylline is also a nonselective competitive inhibitor of phosphodiesterases (PDE) 3 and 4 which results in relaxation of smooth muscle cells in the airways, and this in fact is the main mechanism of theophylline bronchodilation effect [2]. In addition, there are studies suggesting theophylline rule in stimulation of calcium release from intracellular stores and also in IL-10 secretion and apoptosis of inflammatory cells [1].

Being an adenosine receptor antagonist, patients on theophylline, as seen in our case, when in tachycardia state, may be resistant to treatment with adenosine, due to blocked receptors by the theophylline in the serum. Therefore, failure of regular dose treatment with adenosine is not surprising. Adenosine may still be attempted however as there have been case reports of successful SVT treatment with repeated adenosine therapy [4]. It has been known that most common types of arrhythmias associated with theophylline are supraventricular tachycardia (SVT) [5]. This is seen in our patient's case as well. Adenosine, either on initial or repeated dosing, can reverse theophylline induced supraventricular tachycardia [6]. But it should be given with caution in patients with severe pulmonary obstructive disease due to its bronchoconstriction effect. In such cases, selective beta 1 antagonist such as esmolol can safely be used to terminate SVT [7]. Calcium channel blockers such as diltiazem or verapamil can also be used but in caution in patients with hypotension [8]. In our case, given failure of response to initial therapies and patient's hemodynamic instability by hypotension, amiodarone and fluid resuscitation was used with eventual success in stabilizing patient. Amiodarone is an antiarrhythmic medication with known severe potential side effects, and given no prolonged use indication or requirement in our patient, it was not continued beyond the initial bolus administration in our patient.

It is important to recognize the possibility of multifactorial etiology for our patient's presentation. Alcohol and dehydration can also contribute and exacerbate tachycardia. Termination of SVT was therefore most likely multifactorial secondary to spontaneous resolution, fluid resuscitation, and amiodarone therapy. Our patient was recommended to follow-up with cardiology and pulmonary clinics for better guideline-based therapies for his chronic conditions.

## 4. Conclusion

Theophylline and adenosine interactions are well known and used in laboratory settings for years, but clinical cases are rarely reported in literature. This case is significant in showing clinical manifestation of these interactions. Providers' careful medication review not only can aid in timely diagnosis and understanding the underlying pathophysiology of an acute situation but can also help to prevent adverse effects. It is also important to regularly review chronic medications and substitute for any possible newer options that might have less adverse effects compared to older medications.

## Figures and Tables

**Table 1:** Complete blood count, comprehensive metabolic panel, troponin, and theophylline blood test results.

Blood test	Result	Normal range
White blood cell count	4.2 K/MM3	4-11 K/MM3
Red blood cell (RBC) count	3.86 M/MM3 L	4.30-6.00 M/MM3 L
Hemoglobin	12.0 g/dL L	13.5-17.00 g/dL L
Hematocrit	36.8% L	40.0-53.0 L
Mean corpuscular volume	95 fL	78-100 fL
Mean corpuscular hemoglobin	31.1 pg	27.0-34.0 pg
Mean corpuscular hemoglobin concentration	32.6 g/dL	31.0-37.0 g/dL
Red cell distribution width—coefficient of variation	15.6% H	11.0-15.0%
Red cell distribution width—standard deviation	54.4 fL H	38.0-49.9 fL
Nucleated RBC, automated	0%	≤0%
Platelet count	173 K/MM3	130-450 K/MM3
Mean platelet volume	10.1 fL	9.0-12.0 fL
Albumin/globulin ration	1.3	1.0-2.0
Albumin	3.9 g/dL	3.4-5.0 g/dL
Alkaline phosphatase	89 IU/L	40-150 IU/L
Alanine aminotransferase	19 IU/L	10-60 IU/L
Anion gap	12	4-16
Aspartate aminotransferase	38 IU/L	10-50 IU/L
Bilirubin total	1.1 mg/dL	0.2-1.3 mg/dL
Blood urea nitrogen (BUN)	4 mg/dL	8-25 mg/dL
BUN/creatinine ratio	4	10-28
Calcium	9.2 mg/dL	8.3-10.4 mg/dL
Chloride	104 mmol/L	96-110 mmol/L
CO2	21 mmol/L	21-31 mmol/L
Creatinine	0.90 mg/dL	0.60-1.50 mg/dL
Estimated glomerular filtration rate (African descent)	105 mL/min/1.73 m^2^	≥60 mL/min/1.73 m^2^
Glucose	134 mg/dL	65-99 mg/dL
International normalized ratio (INR)	1.0	0.9-1.1
Lipase	24 IU/L	8-78 IU/L
Potassium	3.5 mmol/L	3.5-5.2 mmol/L
Protein, total	7.0 g/dL	6.0-8.0 g/dL
Sodium	137 mmol/L	135-145 mmol/L
Troponin-I, conventional	<0.01 ng/mL	0.00-0.02 ng/mL
Theophylline	4.67 *μ*g/mL	10.00-20.00 *μ*g/mL

## Data Availability

All the data is provided in the case report; please reach out to the corresponding author if any questions.
